# Assessment of the vaccine industry in Iran in context of accession to WTO: a survey study

**DOI:** 10.1186/2008-2231-20-19

**Published:** 2012-08-30

**Authors:** Amir Hashemi Meshkini, Abbas Kebriaeezadeh, Rasoul Dinarvand, Shekoufeh Nikfar, MohammadGafar Habibzadeh, Iman Vazirian

**Affiliations:** 1 Department of Pharmacoeconomics and Pharmaceutical administration, Faculty of pharmacy, Tehran University of Medical Sciences, 16th Azar st, Keshavarz Blvd, Tehran, Iran; 2 Department of Pharmaceutics, Faculty of Pharmacy, Tehran University of Medical Sciences, 16th Azar st, Keshavarz Blvd, Tehran, Iran; 3 Food & Drug Laboratory Research Center, Food & Drug Organization, Ministry of Health & Medical Education, Tehran, 1314715311, Iran; 4 The Department of Law, human science faculty of Tarbiat Modarres University, Kargar Street, Tehran, Iran

**Keywords:** Vaccine, WTO, Iran, WHO

## Abstract

**Background:**

The vaccine industry is one of the most important health-related industries. It can be affected by accession to the World Trade Organization (WTO) because of probable dramatic changes in the business environment. Iran has already initiated accession negotiations.

**Purpose of the study:**

In this paper, we investigate the position of, challenges to, and opportunities for vaccine manufacturing in Iran with regard to accession to the WTO.

**Methods:**

This is a qualitative and cross sectional study. To collect information, we designed a questionnaire and interviewed some of the vaccine industry’s key opinion leaders in Iran. Before the interviews were conducted, the questionnaires were sent to these individuals by email.

**Results:**

According to the interviewees, the country’s main challenges with regard to accession to the WTO are the lack of firm internal intellectual property (IP) rules, not being recognized as pre-qualified by the World Health Organization (WHO), the use of old equipment, and a lack of cooperation with global vaccine companies.

**Major conclusions:**

Iran’s local vaccine industry, with a long history and international reputation that could be used as an advantage, is faced with several challenges, such as problems with keeping up with Current Good Manufacturing Practice (cGMP), a lack of adequate and meaningful investment in research and development (R&D), and limitations on private sector participation in the production of vaccines.

Gradual privatization of the industry, improved international relations, utilization of the R&D power of small hi-tech companies, consistent education of human resources, and modernization of infrastructures and equipment are among the suggested solutions.

## Introduction

Globalization could be defined as the process of making nationalization irrelevant in markets, laws, and politics in the production of common goods, based on comparative advantages [1].

Today, most countries have joined the World Trade Organization (WTO) and some are observer members waiting to start negotiations in the accession process [2]. Iran (the Islamic Republic of Iran) was accepted into this organization in 2005 as an observer member. The Iranian trade regime report was sent to the WTO in 2009, and currently, the first level of negotiation is on the table.

Accession to the WTO most probably affects many aspects of various industries; being familiar with these effects can provide organizations opportunities to deal with the potential challenges that lie ahead. Among these industries, those related to public health, such as the pharmaceutical and vaccine industries, are extremely vulnerable and sensitive: “vulnerable” because they are faced with competition from big international companies inside and outside of the country, and “sensitive” because these industries are related to one of the most important aspects of people's life—their health—especially in developing countries where accessibility to medicines and vaccines has been improved by domestic manufacturing.

The vaccine industry in Iran has a long history. The Razi Serum and Vaccine Research Institute started manufacturing veterinary vaccines in 1929, and soon after, added human products to its agenda [3]. The Institute Pasteur of Iran is the second vaccine manufacturer in Iran. It started in 1920 with the cooperation of the Institute Pasteur of France [4].

With regard to human vaccines, Razi produces measles, polio, MMR, MR, trivalent vaccines, and divalent vaccines. The Institute Pasteur produces BCG and Hepatitis B vaccines.

These two vaccine producers experienced glorious periods in the past. To ensure continued success, the Iran vaccine industry needs to determine its weaknesses and strengths as well as the obstacles and opportunities that lie ahead with regard to WTO accession.

The aim of this study is to assess the probable impacts of WTO accession on the vaccine industry in Iran.

## Method

This is a qualitative cross sectional study to investigate the current situation as well as the upcoming issues affecting the vaccine industry in Iran. To collect data and information, we interviewed some of the key opinion leaders in the Iranian vaccine industry. This group of experts was comprised of representatives from both local vaccine manufacturers and the Ministry of Health. We designed an open questionnaire, including 13 questions that were asked orally.

The questions were classified in 6 groups in terms of the subject:

1- The need for vaccines in Iran in terms of the current health conditions.

2- The vaccine supply in Iran.

3- The effect of international relationships on the vaccine supply in Iran.

4- The vaccine production capacity in Iran in case of crises.

5- The introduction of model countries to the Iranian vaccine industry.

6- The importance of international cooperation in the vaccine industry and Iran’s current status.

7- The current technology level in Iran’s vaccine industry.

8- The current human resources situation in Iran’s vaccine industry.

9- The current situation regarding R&D investment in Iran’s vaccine industry.

10- Public and private sector partnerships in the Iranian vaccine industry.

11- The probable challenges in WTO accession.

12- Iran’s ability to compete in the international vaccine market.

13- Suggested solutions.

In the first question, our aim was to collect information about Iran’s domestic vaccine market and immunization trends in Iran.

In questions 2, 3, and 4, we asked the experts about the capabilities of Iran’s production of necessary vaccines in routine situations and in the event of a crisis.

In questions 5 and 6, we asked about the country’s vaccine manufacturers’ international cooperation and their visions and patterns for the future.

In questions 7, 8, and 9, we were searching for an evaluation of three important factors—the level of technology, human resources, and R&D—in the Iranian vaccine industry.

In question 10, we asked about possession and ownership issues in the vaccine industry in Iran.

Questions 11, 12, and 13 reflect an international market perspective and Iran’s ability to penetrate these markets after WTO accession. We wanted to know about the challenges ahead and the experts’ suggested solutions for overcoming them.

The questionnaires were sent to the interviewees before the interviews.

Among the 16 respondents selected, 11 of the key opinion leaders opted to answer the questions orally. Some of these interviewees only answered some of the questions. Two interviewees preferred to communicate through email, and one of these answered only half of the questions.

## Results

### The need for vaccines in Iran in terms of the current health conditions

The interviewees believed that, regarding routine and traditional vaccines, the immunization coverage in terms of the health conditions (polio has been eradicated, and some diseases, like measles, are expected to be eradicated soon) is adequate. However, the young population and the geographic position of Iran, which neighbors poor countries like Iraq and Afghanistan in terms of health conditions, and illegal immigrants coming from these countries makes Iran vulnerable to the transmission of diseases coming from outside of its borders. Additionally, the large number of foreign trips embarked upon by Iranian citizens, like the Hajj pilgrimage, and communication with people from poor-health-conditioned countries, such as some African countries, are threats to the current domestic situation.

Two of the interviewees believed that the need for vaccines is independent of the prevailing health conditions and is almost constant and contingent upon the population. Another interviewee highlighted the increasing need for vaccines in Iran because of the young population, illegal immigration from neighboring countries (without powerful governments and health systems), and the existence of remote rural areas without adequate health care in some parts of Iran.

### The vaccine supply in Iran, the effect of international relationships, and the vaccine production capacity in Iran in case of crises

Only 9 out of 11 interviewees answered the first question about the vaccine supply in Iran. All of them believed that most of Iran’s vaccine supply is supported by two local manufacturers and, each year, shortages are compensated by import. We were not able to access accurate information about the vaccine market value and market share of Iranian manufacturers, but an estimation by the drug office of the Food and Drug Organization (FDO) from the Health Deputy’s orders for vaccines shows that sales have been 250 to 400 billion Rials annually for the past five years, depending on the year’s requirements. No separate data was available about the value and volume of domestic and imported vaccines.

Ten interviewees answered the second question regarding the effects of international relationships on the vaccine supply. Two respondents thought that international relationships were important and one thought that international relationships would greatly affect the vaccine supply. The other respondents went into detail regarding their opinions: they felt that the main effects of international relationships are on the transfer of technology and access to modern equipment, and that the vaccine supply is not greatly influenced by political issues.

Ten out of 11 interviewees answered the question about Iranian manufacturers’ capacity to supply the needed vaccines in the event of crises. Seven respondents said that, with concentration on the vaccine industry, Iran would be able to cover its domestic needs in crises and extraordinary situations.

### The introduction of model countries and the importance of international cooperation in the vaccine industry

Eleven persons expressed their thoughts on model countries for the Iranian vaccine industry. They listed various countries among their answers: Turkey, Pakistan, Jordan, Egypt, the USA, Canada, India, the European countries, Japan, South Korea, Indonesia, Cuba, and Thailand. Out of these countries, India (seven persons), Indonesia (six persons), and Cuba (four persons) were the most mentioned. Four out of the 11 key opinion leaders believed that developed countries like the USA, Japan, and Canada should be used as models, while the others emphasized the significance of economic similarity between Iran and the model countries. They selected their models from developing countries with WHO prequalification.

Regarding the importance of international cooperation between vaccine companies and the current situation in Iran, 9 out of the 10 persons who answered this question pointed to the importance of this relationship with certainty. Seven out of 11 interviewees found Iran’s current situation undesirable and two of them did not respond.

### The current situation in Irans vaccine industry regarding technology, human resources, and R&D

Regarding the level of technology, 10 out of 11 respondents mentioned difficulties in accessing new equipment. Three of them found that the position of the Institute Pasteur was superior to the Razi Institute because it has newer facilities. Regarding the level of technology in vaccine manufacturing, two out of seven respondents said that Iranian methods of vaccine production are current and are based on international protocols, and the only problem is accessing new equipment; however, four of these seven felt that the vaccine technology level is undesirable and that Iran is not in a good global position. Nine respondents believed that Iran has achieved significant improvements in biotechnology in recent years but that these achievements have not yet been fully exploited. All of those with positive outlooks regarding the level of technology were representatives from Iranian vaccine manufacturers.

Four out of 10 interviewees who answered the question regarding the human resource situation called it satisfactory and the others mentioned some weaknesses, like the shortage of well-educated persons and experts in the industry, the lack of industry skills in university graduates, problems in consistent education within the industry, and the lack of cooperation between Iranian and foreign vaccine makers in the field of human resources enhancement.

Nine persons responded to the question about R&D. All of them called the investment of local Iranian manufacturers in R&D negligible in comparison with international norms. Five persons believed that the Institute Pasteur’s investment in R&D is superior to that of the Razi Institute. Low levels of investment, non-applicable and non-targeted research, and problems in R&D management were the main issues presented by the interviewees.

### Public and private sector partnerships

All 11 persons who responded to this question stated that the private sector does not have a role in vaccine production in Iran. Three pointed out the tendency of the private sector to engage in vaccine production. The extent of private sector participation in vaccine development and production in Iran is very small and limited to a few companies that import vaccines ordered by the government. One interviewee believed that the only route of entry into this market is focusing on exportation and foreign markets. Some of the experts believed that most of the problems in Iran’s vaccine industry are related to its public structure.

### Irans ability to compete in the international market, the probable challenges to WTO accession, and suggested solutions

For the question about Iran’s ability to compete in the international vaccine market, two experts responded positively and three believed that it is not possible for Iran to compete. The others believed there is a provisional possibility. Four interviewees stated that this ability depends on obtaining WHO prequalification and one person stated that this ability depends on using current capacities and expanding upon them.

Ten out of 11 interviewees commented on the probable challenges to the vaccine industry during WTO accession. One of them believed that joining the WTO would be detrimental to the vaccine industry in Iran, but he did not provide any support for his opinion. Another respondent believed that IP issues are the main challenges. Other challenges mentioned included outdated equipment and political challenges, like sanctions and lack of international cooperation. Not being prequalified (one person) and weaknesses in state-run industries (one person) were the other ideas. Two believed there are no challenges, as the country is able to overcome any obstacles by using WTO flexibilities.

These key opinion leaders believed in the effectiveness of the following solutions: privatization of the industry (four persons), consistent education for employees (three persons), allowing private sector involvement along with the government in production (two persons), using the capacity of small science-based companies in R&D and related areas (two persons), the improvement of international cooperation in the vaccine industry (one person), the resolution of political challenges (one person), and determination and implementation of IP rights in Iran with the help of foreign consultants (one person).

## Discussion

### Need for vaccines in Iran in terms of the current health conditions

Vaccines needed for immunization programs in Iran (EPI) include BCG, DTP, HepB, polio, and MCV (see Table 1). The influenza, rabies and HPV vaccines (all imported from other countries) are available on the market but not in the EPI and there is still a clear lack of availability of Haemophilus influenza type B (Hib) and rotavirus vaccines in EPI. According to an internal study, the prevalence of Hib carriers is 7.6%, similar to other developing countries, yet this vaccine is still not part of the program [5]. The government intends to include these two vaccines in the program; this is dependent both on adding them to the Iranian drug list and the ability of vaccine producers to produce them, which has not been the case so far. It should be emphasized that Iran has already adopted a National Drug List (NDL), which is selected by the Iran Drug Selecting Committee (IDSC). All drug supply management process, including registration, procurement, inspection, quality control, and post marketing control, can be handled by the IDSC for a drug that is accepted for inclusion on the NDL [6].

**Table 1:** Immunization schedule of expanded program of immunization program in Iran, 2010

**Age of vaccination**	**Type of vaccine**
At birth	BCG, OPV, Hep B
2 month	OPV, DTwP, HepB
4 month	OPV, DTwP
6 month	OPV, DTwP, Hep B
12 month	MMR
18 month	OPV, DTwP, MMR
6 years	OPV, DTwP

### The vaccine supply in Iran, the effect of international relationships, and the vaccine production capacity in Iran in case of crises

It seems there is no clear vision in Iran regarding the vaccine supply in the future. The world’s approach toward combo vaccines and changes in the type of vaccines, such as the expanded use of inactivated polio vaccine (IPV) instead of oral polio vaccine (OPV) and the probable eradication of some diseases within the country in the coming years, could significantly affect Iran's vaccine industry.

With the advent of new diseases and phenomena like bioterrorism, the capacity for vaccine production has become increasingly important. Some developed countries, such as the United Kingdom [7] and the United States [8] have invested in this sector to assure their ability to control such threats. The Iranian vaccine industry has illustrated its abilities to supply domestic need in crises; the main example of this is its performance during the imposed war with Iraq. As most key opinion leaders stated, they are not concerned about crises, however, the country must continually increase its capacity.

### The introduction of model countries and the importance of international cooperation in the vaccine industry

There was general agreement among those interviewed regarding international cooperation for growth in this industry. Today, the importance of collaboration by vaccine makers in developing countries has become clear. The LG Company in Korea, which has obtained WHO prequalification, is establishing strategic cooperation in the field of R&D with large vaccine companies [9]. According to reports published by the local government, such cooperation by the Institute Pasteur for production of pentavalent vaccine and other biotechnology products with countries such as Cuba, India, Malaysia, and France has already been enacted [10], although most of the interviewees found these partnerships to be very limited, especially in the case of the Razi Institute.

### The current situation of Irans vaccine industry regarding technology, human resources, and R&D

According to the interviewees, there are lesser problems in access to technology for traditional and well-established vaccines; however, because of the current and upcoming changes in the methods of producing these vaccines, Iran’s vaccine industry needs to be updated by acquiring new technologies for surviving international competition. In the field of newer vaccines, access to equipment and devices, especially access to technology, is influenced dramatically by political issues.

It seems that despite highly educated professionals in this industry, the human resources situation in Iran is far from ideal because of the lack of regular targeted training and the need for updated knowledge and information, which could be because of governmental structure and political problems and challenges.

The best source for R&D work on the diseases of developing countries is the countries where the diseases are endemic. The Commission on Health Research for Development in 1990 showed that only 10% of investment in health research is allocated to 90% of the diseases and health problems of the world. This is called the 10/90 gap. Children in developing countries do not benefit from advances in the field of vaccines in richer countries [11]. Iran could recognize the vaccine market in developing and less developed countries and manage research and development investments toward their needs to be able to use these opportunities in the future. By following the current approach in the management of R&D in Iran, the vaccine industry will not be able to participate in global markets or even cover domestic needs. According to an internal report by the Razi Institute [12], the rate of capital invested by the Razi Institute in R&D in the annual budget is only 2%; in light of the large areas of activity in this institute in the field of vaccines and serums (human and animal), this seems to be a very limited amount.

### Public and private sector partnerships

The Ministry of Health is responsible for supplying the needed vaccines and planning the vaccination programs. In Iran, the government is involved in both producing and supplying vaccines. The Institute Pasteur is under supervision of the Ministry of Health and the Razi Institute operates under the Ministry of Agriculture. However, because of being regarded as a strategic industry for Iran in the area of national security, the possible privatization of these two vaccine makers is not imminent. As previously stated, it seems that the presence of the private sector along with the public sector would help the vaccine industry to overcome many problems, such as efficiently maintaining and strengthening human resources, managing and optimizing targeted areas of R&D capacities, and the provision on opportunities to establish a presence in international markets. Several factors impede the private sector from becoming involved in vaccine production. The only purchaser for the EPI vaccine is the government and because both vaccine manufacturers belong to the government, there is not an opportunity for the private sector to become involved in such a market. The government prefers to have complete authority in vaccine production and supply so that it can assure a consistent supply regardless of gain or loss (which is the main incentive for the private sector) at different times and in different situations.

### Irans ability to compete in international markets, the probable challenges to WTO accession, and suggested solutions

Most expert interviewees feel that the first step in moving toward international market status is obtaining WHO prequalification for the country’s vaccines. Today, many countries are faced with several problems in supplying essential vaccines. Concerns in this regard increased after the implementation of IP rules and discussions related to world trade [13]. But some vaccine-related agencies, such as the United Nations Children's Fund (UNICEF) and the Pan American Health Organization (PAHO), and government–private cooperatives, like the Global Alliance for Vaccines and Immunization (the GAVI Alliance), are working to help these countries. GAVI, for example, aims to increase the economic attractiveness of the market by stimulating vaccine demand in developing countries, as well as strengthening infrastructure and ensuring their ability to procure such products [14]. UNICEF also purchases about 55% of the world’s vaccines [15]. The World Health Organization grants prequalification to ensure that UN agencies buy quality, safe, and efficacious vaccines. According to internal news, Iran’s National Regulatory Authority (NRA) recently gained WHO prequalification, but many problems concerning cGMP compliance still require resolution. Unfortunately, due to lack of sufficient investment, it is not possible to upgrade the facilities and equipment at the Razi Institute to comply with the principles of cGMP. However, most interviewees believed that it will be easier to obtain these standards by the Institute Pasteur’s new facility (where the Hep B vaccine is manufactured).

The future of the vaccine industry after accession is very important because it is directly related to the country’s public health. Therefore, it is necessary to understand the WTO rules affecting this industry. Three agreements— “Trade Related Aspects of Intellectual Property Rights (TRIPs),” “Subsidies and Compensatory Measures,” and “Government Procurement Agreement (GPA)”—could be associated with this industry and must be carefully considered. Here, we review one of the most important agreements: TRIPS.TRIPs: Intellectual property right is considered as an asset by companies which could be seen as patents, distinctive signs, copyright, industrial designs and trade secrets [16]. In pharmaceuticals, patent rights cause a monopoly for patent holders by preventing others from using a particular invention for a specified period of time [17]. Nevertheless, to resolve the concerns of developing countries [18], the TRIPs agreement offers some flexibility for governments in providing their social goals. Transition periods, compulsory licenses, and parallel importation are from such flexibilities [19]. But in terms of being influenced by TRIPs, there are some differences between vaccines and other pharmaceutical products. The most important difference is the complexity of the processes used to manufacture biological products like vaccines. There are considerable scientific techniques and secrets (know-how) in the manufacturing processes of such products that companies prefer to maintain in-house as trade secrets and which will not be disclosed; therefore, compulsory licenses would not be all that useful [20]. Another issue is that it has become usual to patent one or more of the vaccine components, such as adjutants [21]. In a study conducted in 2005, the World Health Organization showed that most suppliers of vaccines in developing countries have not considered that IP plays an important role in vaccine R&D [22]. Iranian drug policy makers should also ensure efficacy, quality, and safety measures by full adherence to Iranian national drug policy rules [23]. These issues should be paid adequate attention before proceeding to WTO accession.

## Conclusions

Both strengths and weaknesses were found in the Iranian vaccine industry, although the weaknesses outweigh the strong points. Considering the fact that it is a large market in relation to its young population and large area as well as its specific geographical location (associated with the war-torn countries without strong health systems and appropriate levels of health), Iran is experiencing an ever-increasing need to supply vaccines; the increasing need and market could be an opportunity for this industry, even on a domestic level. The long history and reputation of Iran’s local vaccine industry could be considered advantages. However, this industry is faced with some weaknesses, such as non-compliance with cGMP, a lack of adequate and meaningful investment in R&D, a lack of mutual reliance of the government on the private sector in vaccine production, and the problems and political challenges that have hindered the development of this industry. According to most of the key opinion leaders, the most significant problem in Iran’s vaccine industry has roots in its government ownership, which cannot be quickly solved.

Some useful solutions for improving the position of this industry in international competition include: a) privatization of current vaccine manufacturers (in the long-term) and relying on private vaccine producers to come into the field, b) improving international relations and cooperation with the large vaccine manufacturers in the world, c) utilizing small knowledge-oriented companies with the aim of fueling R&D, d) consistent education for employees, e) investment in order to modernize the old infrastructure of the Razi Institute regarding compliance with the principles of cGMP to achieve international quality standards to facilitate WHO prequalification. For the implementation of these strategies and to minimize the negative effects of implementing WTO agreements like GPA, TRIPs, and subsidies and compensatory measures, the role of negotiation teams in the accession process is extremely crucial. These teams should be familiar with the status of the vaccine industry. Achieving agreement among the industry’s key opinion leaders about the future of this industry in the process of WTO accession can facilitate the negotiations. It is obvious, then, that recruiting professional experts in this field is necessary for this purpose.

## Competing interest

The authors declare that they have no competing interests.

## Author’s contribution

AHM: literature review, designing the questionnaire and conducting interviews. AK: Designing the study method, selection of interviewees and monitoring the various stages of study. RD: Selection of interviewees, monitoring various stages of study. SN: Interpreting the results and final revision of the manuscript. MGH: providing advices in legal issues. IV: Participation in literature review and designing the questionnaire. All authors read and approved the final manuscript.
